# Clinical Positioning and Implementation of a Deep-Learning Retinal Biomarker (Reti-CVD) for Cardiovascular Risk Stratification: A Narrative Review

**DOI:** 10.3390/jcm15145649

**Published:** 2026-07-18

**Authors:** Junseung Rho, Sung-Goo Kang, Se-Hong Kim, Sang-Wook Song

**Affiliations:** Department of Family Medicine, St. Vincent’s Hospital, College of Medicine, The Catholic University of Korea, Seoul 16247, Republic of Korea; mediart@catholic.ac.kr (J.R.); iron1600@catholic.ac.kr (S.-H.K.); fmssw@catholic.ac.kr (S.-W.S.)

**Keywords:** retinal imaging, deep learning, cardiovascular disease risk, oculomics, AI-based medical device, risk stratification, clinical utility, primary prevention

## Abstract

Cardiovascular disease (CVD) prevention depends on accurate risk stratification before symptoms develop. Standard tools such as the Pooled Cohort Equations, QRISK3, and SCORE2 require laboratory data and are less informative in borderline-risk individuals, creating a role for accessible adjuncts. Retinal imaging directly visualizes the systemic microvasculature, and deep-learning oculomics may provide complementary risk information. Reti-CVD generates a three-tier classification from a retinal photograph and is among the more extensively validated retinal-AI tools. This narrative review evaluates its clinical positioning and implementation as an exemplar rather than a product endorsement, organizing evidence by cohort, comparing the approach with established scores and subclinical atherosclerosis markers, and considering implementation, regulation, and equity. RetiCAC was trained using coronary artery calcium as a surrogate label; subsequent Reti-CVD studies included UK Biobank, Singapore SEED, and CMERC-HI. Reported discrimination was approximately 0.75 by the Harrell C-index, with modest reclassification improvement, particularly in borderline-risk groups. As the commercial product DrNoon for CVD, the tool holds marketing authorization from Korea’s Ministry of Food and Drug Safety (MFDS) and, according to the manufacturer, CE certification under the EU Medical Device Regulation (MDR); in Korea it entered outpatient practice through a time-limited non-covered (out-of-pocket) assessment-deferral pathway, and it has not yet received US FDA authorization. Most evidence originates from one research group and one commercial algorithm, and no randomized or outcome-based study has shown that Reti-CVD-guided care improves clinical outcomes. These observational findings remain hypothesis-generating rather than evidence of established clinical utility. Reti-CVD is therefore best regarded as a non-invasive risk enhancer for borderline/intermediate-risk reclassification, not as a tool of established clinical utility; independent validation, intervention trials, and cost-effectiveness and reimbursement evidence are needed before broad integration.

## 1. Introduction

### 1.1. Current State and Unmet Need in Cardiovascular Risk Assessment

Cardiovascular disease remains the single leading cause of death and disability worldwide, and its burden continues to grow with population ageing and the rising prevalence of metabolic disorders such as obesity and diabetes [1]. The foundation of primary prevention is to quantify an individual’s 10-year risk in the asymptomatic stage and thereby select those who would benefit from interventions such as statins or antihypertensive therapy. To this end, well-validated multivariable risk scores—the PCE in the United States, QRISK3 in the United Kingdom, and SCORE2 in Europe—are embedded in clinical guidelines and generally use specific 10-year risk thresholds (e.g., 7.5% or 10%) to guide decisions about pharmacological intervention [2,3,4,5,6].

However, these standard tools all require, in addition to age, sex, blood pressure, smoking, and diabetes, the results of lipid testing such as total cholesterol. Although such laboratory testing is inexpensive and widely available in routine practice, it presupposes blood sampling and laboratory infrastructure, so the applicability of these scores may be constrained for non-laboratory-based opportunistic screening and in primary-care or community settings with limited access to such testing. Moreover, many individuals do not fall into clearly low- or high-risk categories but into the intermediate/borderline range, precisely where treatment decisions are most ambiguous. To address this, recent guidelines recommend considering so-called “risk-enhancing factors” such as the coronary artery calcium score, family history, and high-sensitivity C-reactive protein [5,6]. Even so, many of these additional markers again require blood draws, imaging, or specialized equipment, leaving an unmet need for an adjunct marker that is both non-invasive and highly accessible.

### 1.2. Subclinical Atherosclerosis Imaging and Its Limitations

One of the most powerful single markers for risk reclassification is the coronary artery calcium (CAC) score measured by cardiac CT. CAC is itself non-invasive, directly quantifies subclinical atherosclerosis, and has demonstrated robust predictive value and reclassification benefit beyond traditional risk factors in multiethnic cohorts; it is accordingly endorsed in guidelines as a decision aid for selected intermediate-risk individuals [5,7,8]. Its broader use for population-level screening is, however, tempered by practical considerations such as the need for dedicated CT equipment, low-dose radiation exposure, cost, and interpretation infrastructure. Other subclinical markers such as carotid intima-media thickness (CIMT) and brachial-ankle pulse wave velocity (baPWV) also have their own constraints of operator dependence or standardization. Consequently, there is interest in complementary markers that are highly accessible and carry biological information about atherosclerosis, ideally without requiring radiation or specialized imaging facilities.

### 1.3. The Retina as a Window to the Systemic Vasculature and the Rise of Oculomics

The retina is one of very few anatomical sites at which the microvasculature can be observed directly and non-invasively in vivo. Retinal arteriolar narrowing, venular dilatation, and arteriovenous nicking reflect systemic hypertensive and atherosclerotic changes, and large epidemiological studies have repeatedly reported associations with coronary heart disease, stroke, and cardiovascular mortality [9,10,11,12]. However, traditional manual vessel measurement is labor-intensive and limited in reproducibility, restricting clinical use.

Recent advances in deep learning have opened the field of oculomics, in which systemic information such as age, blood pressure, and smoking is predicted from subtle retinal patterns imperceptible to humans; the rapid growth of this field has been summarized in recent reviews [13,14,15,16]. In ophthalmology, deep learning has already undergone clinical validation and regulatory clearance for tasks such as diabetic retinopathy screening [17,18], demonstrating that retinal-image-based algorithms can be integrated into clinical practice. In the cardiovascular domain as well, automated retinal vessel measurement [19] and risk-factor/risk prediction models [13,20] have been reported in succession, and the emergence of retinal foundation models indicates that the field is maturing rapidly [21].

### 1.4. Aim, Scope, and Framing of This Review

Within this landscape, Reti-CVD merits independent appraisal for two reasons. First, it has been validated in multiple external cohorts and in a validation study supporting regulatory authorization, so the quantity and breadth of evidence are comparatively substantial [20,22,23,24]. Second, while most oculomics algorithms remain at the research stage, Reti-CVD is among the relatively few that have progressed through a regulatory pathway toward clinical application [24,25,26]. For terminology, throughout the Introduction “Reti-CVD” denotes only the clinical three-tier risk-stratification index evaluated in subsequent validation studies; “RetiCAC” denotes the CAC-trained precursor, and “DrNoon for CVD” denotes the commercial device (see Box 1).

Box 1 **Terminology: RetiCAC, Reti-CVD, and the Commercial Product (DrNoon).**

**Key distinctions**

**RetiCAC—**a deep-learning score trained using coronary artery calcium (CAC) as the ground-truth label to predict the probability of CAC presence or CAC-related risk from a retinal photograph. It is a development/research-stage concept developed and validated by Rim et al. (2021) [20]. Its training target is CAC.

**Reti-CVD—**the retinal CVD risk-stratification index assembled with clinical application in mind. It was used as the validation target in subsequent external-validation and regulatory-supporting validation studies, with output as low/moderate/high three-tier risk groups [22,23,24]. Its intended use is risk stratification toward clinical application.

**DrNoon for CVD (Dr. Noon CVD)**—the product name of the commercialized, authorized software medical device based on Reti-CVD (manufacturer: Mediwhale, Seoul, Republic of Korea). Regulatory details are addressed in Section 6 [25,26,27].

**A note on the relationship.** The three names lie on a continuum, but their training targets (CAC probability vs. CVD risk stratification) and intended uses (development vs. clinical application) are not entirely identical. Because the public literature does not explicitly describe the algorithmic identity of RetiCAC and Reti-CVD (whether weights are identical, or whether recalibration occurred), this review does not assert it and instead specifies the output index validated by each study in Table 1. Accordingly, the evidence from the RetiCAC stage supports the conceptual validity of Reti-CVD but should not be equated with the clinical evidence for Reti-CVD itself.

Two framing choices follow from this. First, we treat Reti-CVD as an exemplar—currently among the most extensively externally validated and regulatory-advanced instances—of the broader approach of retina-based deep-learning CVD risk stratification, rather than as an endorsement of a specific commercial product; competing and complementary approaches are considered explicitly in Section 4.5. Second, because much of the core evidence originates from a limited set of investigators and a single commercial algorithm, we foreground this concentration throughout and treat it as a central caveat (Section 4 and Section 7). Accordingly, the title and emphasis of this review concern clinical positioning and implementation rather than demonstrated clinical utility, which the current evidence does not establish. To reduce the risk of product-specific overinterpretation, generalizable clinical principles are separated from product-specific regulatory or market-access claims, and the latter are interpreted only when supported by publicly verifiable sources.

**Figure 1:**
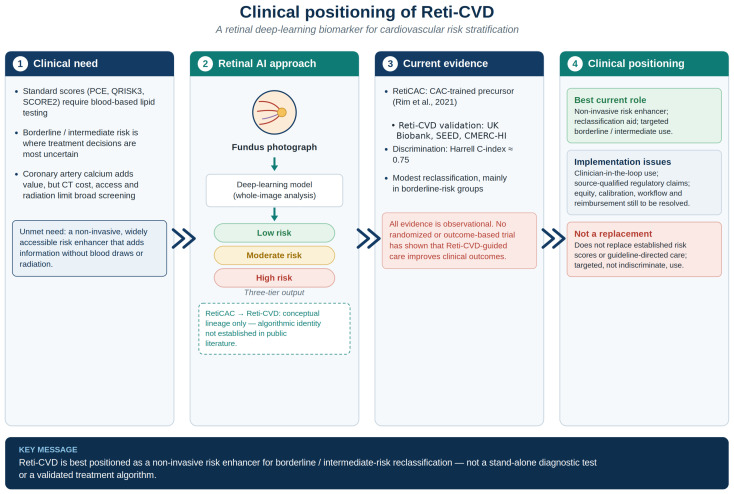
Overview of the clinical need, the retinal AI approach, the current evidence, and the clinical positioning and implementation of Reti-CVD. The arrow from RetiCAC to Reti-CVD denotes conceptual lineage; algorithmic identity was not established in the public literature. SEED, Singapore Epidemiology of Eye Diseases. The figure summarizes evidence categories and implementation considerations rather than a validated clinical workflow [20].

This review is deliberately differentiated from a prior scoping review that technically surveyed retinal deep-learning CVD prediction as a whole [28]. Rather than cataloguing algorithms, it uses a single, comparatively well-characterized exemplar to critically and integratively appraise (i) the level and consistency of the evidence, (ii) the clinical position of the approach relative to existing risk scores and subclinical atherosclerosis markers, and (iii) real-world implementation, regulation, and equity. It is a narrative review that does not follow the format of a systematic review; it therefore emphasizes interpretive synthesis of the core primary evidence and contextual literature rather than exhaustive collection of all relevant studies. A schematic overview of the clinical need, the retinal deep-learning approach, the current evidence, and the resulting clinical positioning of Reti-CVD that organizes this review is provided in Figure 1.

## 2. Literature Search Strategy

For this narrative review, we searched PubMed/MEDLINE and PubMed Central (PMC) and supplemented these searches with targeted searches of publisher platforms and official or quasi-official sources through 10 July 2026. The main search strings and keyword combinations were the following: “Reti-CVD,” “RetiCAC,” “DrNoon for CVD,” “retinal deep learning cardiovascular risk,” “retinal photograph coronary artery calcium deep learning,” “oculomics cardiovascular,” “retinal fundus image cardiovascular prediction,” and “AI software as a medical device cardiovascular risk.” Reference lists of key primary studies and relevant reviews were also screened to identify additional contextual literature.

During revision, PubMed/MEDLINE and PMC were researched for all publication years available through 10 July 2026 using the Boolean concept: (“Reti-CVD” OR RetiCAC OR “DrNoon for CVD”) OR ((retina* OR fundus OR oculomics) AND (“deep learning” OR “artificial intelligence”) AND (“cardiovascular risk” OR “coronary artery calcium” OR “cardiovascular event*” OR “hypertensive retinopathy”)). Eligible records were human studies reporting algorithm development or validation, cardiovascular outcomes, or clinically relevant implementation; animal studies, conference abstracts, preprints, studies without a cardiovascular target, while purely technical reports without clinical relevance were excluded. The original exploratory screening log was not prospectively retained, so exact record counts could not be reconstructed reliably and were not retrospectively estimated.

The evidence base was organized into three prespecified categories. First, studies that developed, validated, or clinically evaluated Reti-CVD or its direct precursor RetiCAC were treated as the core evidence. Second, studies of adjacent retina-based deep-learning or oculomics approaches, conventional cardiovascular risk scores, and subclinical atherosclerosis markers were included as contextual evidence when they informed clinical positioning. Third, implementation, regulatory, and reimbursement materials were included only when they came from peer-reviewed publications, public regulatory databases, government or statutory sources, or manufacturer materials that were clearly identified as such. Product-specific claims were not generalized beyond the source in which they appeared.

Because this was a narrative review, we did not follow PRISMA procedures, register a protocol, perform duplicate screening, or undertake meta-analysis. We did, however, conduct a concise structured appraisal of the core validation studies using domains adapted from PROBAST for prognostic/prediction studies and QUADAS-2 for the cross-sectional agreement study (participants or patient selection, predictor or index test, outcome or reference standard, and analysis or flow) [29,30]; the findings are reported descriptively in Table 1 without a summary score. The search was intended to capture the core primary evidence and high-relevance contextual sources rather than exhaustively enumerate all retinal-AI cardiovascular studies. Because no protocol was registered and screening was not performed independently in duplicate, the contextual evidence may overrepresent readily retrievable or positive studies and underrepresent negative or less visible approaches.

## 3. Technical and Biological Background

### 3.1. Pathophysiology Linking the Retinal Microvasculature and Systemic Cardiovascular Risk

The retinal circulation shares developmental and structural characteristics with the cerebral and systemic microcirculation, and reflects, relatively early, the changes of endothelial dysfunction, atherosclerosis, and microvascular remodeling caused by hypertension, dyslipidemia, and diabetes. Retinal arteriolar narrowing is known to be associated with elevated blood pressure and incident hypertension, as well as venular dilatation with inflammatory and metabolic abnormalities [10]. Individual-participant meta-analysis has confirmed that retinal vessel caliber is associated with event risk such as stroke [11], and long-term follow-up cohorts have reported that vessel caliber predicts cardiovascular outcomes [12]. This evidence supports the biological plausibility of the retina as a surrogate marker of systemic vascular health. However, the effect size of any single parameter is generally moderate, and it has long been noted that clinical use requires an approach that integrates multiple sources of information.

### 3.2. Evolution of Retinal Image Analysis: From Manual Measurement to Deep Learning

Early studies quantified specific parameters such as vessel caliber using semi-automated software, but this was labor-intensive and subject to inter-observer variability. Deep learning advanced this in two directions. One automates and standardizes vessel measurement itself, showing agreement comparable to expert reading and demonstrating that measured caliber is associated with risk factors and events [19]. The other is an end-to-end approach that predicts systemic information directly from the raw image without explicit measurement, attracting attention for predicting even information—age, sex, smoking, blood pressure—not previously thought legible from the retina [13]. Reti-CVD belongs to the latter category, learning risk signals from the whole image rather than via explicit vessel measurement.

### 3.3. From RetiCAC to Reti-CVD: Development Rationale and Output

The direct origin of Reti-CVD lies in two stages of prior work (see Box 1). First, the investigators developed and validated a deep-learning algorithm to predict systemic biomarkers from retinal photographs using multinational data [31]. They then trained RetiCAC to predict the probability of CAC presence from retinal photographs, using the clinically validated risk marker CAC score as the ground truth [20]. Attempts to predict a high CAC score from retinal photographs have also been reported by other groups [32]. Stratifying RetiCAC into tertiles yielded event-prediction performance comparable to CT-measured CAC, and the refinement of this risk-stratification system for clinical use is Reti-CVD. Choosing CAC as the ground truth is reasonable in that it adopts a pathophysiologically meaningful target—atherosclerotic burden—as the learning signal. However, CAC is itself a surrogate of subclinical atherosclerotic burden rather than a patient-important endpoint such as myocardial infarction or cardiovascular death. Training a retinal model on a CAC-related label therefore introduces a second inferential layer (retinal image → predicted CAC-related phenotype → future clinical event), and error or population-specific calibration at either link may weaken clinical validity. Event-based validation partly addresses this limitation, but agreement with CAC cannot establish that model-guided care improves outcomes.

From a clinician’s perspective, what matters is not the internal weights but the output. Reti-CVD takes a standard fundus photograph as input, produces a risk score, and stratifies it into three tiers (low/moderate/high). In other words, a single retinal photograph yields a three-tier CVD risk-stratification result without blood sampling. As emphasized in Box 1, however, the evidence obtained at the RetiCAC stage and that obtained at the Reti-CVD stage may concern different output indices and are therefore interpreted separately. To be explicit, whether RetiCAC and Reti-CVD share identical model weights has not been definitively established in the public literature; the current evidence therefore supports a conceptual lineage between the two rather than strict algorithmic equivalence, and findings obtained with one index should not be assumed to transfer directly to the other.

### 3.4. Methodological Considerations

Several methodological caveats apply to interpreting deep-learning risk models. First, because model performance depends on the population, device, and image-quality distribution of the training data, the match between the validation and application populations is important. Second, how well a model trained on a surrogate ground truth such as CAC predicts actual clinical events requires separate outcome-based validation. Third, transparency and reproducibility improve when prediction-model reporting follows standard guidelines such as TRIPOD+AI [33]. Fourth, the thresholds for the risk score (the low/moderate/high boundaries) may be optimized in a particular cohort and may require reproduction or recalibration in other populations. These considerations form the basis for the evidence appraisal and the discussion of limitations that follow.

## 4. Clinical Evidence: Organized by Cohort and Context

The key validation studies of Reti-CVD and the direct precursor algorithm (RetiCAC) are summarized in Table 1 by output index, study type, study population, comparator, and key findings. Note that “study type” describes each study’s design and does not assign a hierarchical level of evidence (e.g., Level I–IV). All evidence in Table 1 is observational (prognostic or diagnostic-accuracy) and does not include randomized controlled trials or intervention evidence demonstrating improved clinical outcomes. Table 1 also provides a structured, domain-based, critical appraisal informed by PROBAST and QUADAS-2 [29,30].

**Table 1:** Summary and structured critical appraisal of key validation studies of Reti-CVD/RetiCAC.

Study/Output	Design/Population	Main Findings	Structured Critical Appraisal
Rim et al., 2021 [20] RetiCAC	Development plus multi-cohort external validation; Korea, Singapore, and UK Biobank; 216,152 fundus photographs	CAC-trained score; tertiles predicted fatal CVD events and improved reclassification when added to PCE, particularly in borderline/intermediate-risk groups.	Strengths include scale and multinational testing. Main concerns are health-screening derivation, a surrogate CAC training target, validation largely within the same research ecosystem, and uncertain transportability across populations/devices; clinical impact was not tested.
Tseng et al., 2023 [22] Reti-CVD	External prognostic validation; UK Biobank; *n* = 48,260	Independent association with CVD events; modest increases in C-statistic when added to QRISK3; proposed for borderline QRISK3 (7.5–10%).	Large external cohort, but UK Biobank selection limits representativeness and the three-tier cut-offs were optimized in the same cohort, creating potential optimism. Calibration, transportability, and clinical impact remain unestablished.
Yi et al., 2023 [23] Reti-CVD	Cross-sectional agreement study; UK Biobank plus Singapore SEED; *n* = 55,070	Substantial agreement with PCE-, QRISK3-, and modified FRS-defined intermediate/high-risk groups without blood testing.	The reference standards were the calculated risk scores rather than the clinical outcomes. Agreement therefore cannot establish prognostic validity or patient benefit, and the threshold transportability to other populations remains uncertain.
Lee et al., 2024 [24] Reti-CVD	Retrospective single-center prognostic validation; Korean CMERC-HI; *n* = 1106	Risk groups were associated with CVD events; high-risk HR 3.56 after inclusion of CAC, CIMT, and baPWV; Harrell C-index 0.751.	The regulator-facing analysis used a prospectively assembled cohort, but only 33 events occurred. The single-center sample, limited event count, and wide uncertainty constrain robustness; no independent prospective impact trial was performed.
Syed et al., 2025 [34] Separate model	Adjacent prognostic evidence; type 2 diabetes cohort; *n* = 6127	Retina-predicted risk was associated with MACE and added information when combined with PCE and polygenic risk score.	This supports the broader retinal-AI concept only. The algorithm, population, and target differ from Reti-CVD, so the study cannot be treated as direct validation of Reti-CVD.

Abbreviations: CAC, coronary artery calcium; CIMT, carotid intima-media thickness; baPWV, brachial-ankle pulse wave velocity; PCE, Pooled Cohort Equations; FRS, Framingham Risk Score; HR, hazard ratio; MACE, major adverse cardiovascular event; C-index, concordance index. The appraisal was adapted from PROBAST for prognostic/prediction studies and QUADAS-2 for the cross-sectional agreement study, covering participant selection, predictor/index test, outcome/reference standard, and analysis/flow [29,30]. Because the designs and targets differed, findings are descriptive and no summary score was assigned. Syed et al. (2025) [34] used a separate algorithm and is included only as contextual evidence.

### 4.1. Algorithm Development and Ground-Truth Validation (RetiCAC)

At the development/internal-validation stage, RetiCAC was trained and validated on more than 216,000 retinal photographs from Korea, Singapore, and UK Biobank. Tertile risk stratification showed a dose–response relationship with fatal CVD events and, when added to the PCE, significantly improved reclassification in intermediate- and borderline-risk groups [20]. The significance of this stage is that risk stratification reflecting atherosclerotic burden is possible from retinal photographs alone, and that such stratification can add value to existing scores. However, the output index for this evidence is RetiCAC and, strictly, constitutes the conceptual foundation rather than the clinical evidence for Reti-CVD.

### 4.2. External Validation in a General Population (UK Biobank)

In the UK Biobank, a large general-population cohort (~48,000), Reti-CVD was independently associated with CVD risk, independent of QRISK3 (adjusted HR 1.41). When Reti-CVD was added to QRISK3, the C-statistic improved consistently and modestly in the non-statin, stage-1 hypertension, and middle-aged cohorts, and its potential as a risk-enhancing tool was suggested particularly in the clinically ambiguous borderline-QRISK3 range (10-year risk 7.5–10%) [22]. The absolute improvement is small, but it has practical implications as added information are obtained non-invasively. The output index in this study is Reti-CVD, and it is central to evaluating the incremental value over standard scores in a general population.

### 4.3. Classification Agreement with Existing Risk Scores

A cross-sectional study using UK Biobank and the Singapore SEED study evaluated how well Reti-CVD identifies the intermediate-/high-risk groups defined by existing standard scores (PCE, QRISK3, modified FRS). Against the PCE, it reported sensitivity 82.7%, specificity 87.6%, positive predictive value 86.5%, and negative predictive value 84.0%, indicating substantial agreement with the risk classification of standard scores even without blood tests [23]. This suggests potential use as a first-line screening tool in settings where blood sampling is difficult. However, given the cross-sectional design, this study demonstrated “classification agreement with standard scores” rather than event-prediction ability per se—a point to bear in mind.

### 4.4. Validation Supporting Regulatory Authorization (CMERC-HI)

In a validation study using the Korean CMERC-HI cohort—reported under the title “Pivotal trial of a deep-learning-based retinal biomarker” [24]—Reti-CVD risk groups showed a significant dose–response association with CVD events (HR trend 2.02). Even when the subclinical atherosclerosis reference markers CAC, CIMT, and baPWV were included together in a single model, Reti-CVD retained an independent association (high-risk HR 3.56), and discrimination was reported as a Harrell C-index of 0.751 [24]. Notwithstanding the original title, this study is, in design, a validation analysis of an observational single-center cohort rather than a randomized trial; we therefore refer to it as a validation study supporting regulatory authorization. Given that it was used as supporting evidence in the Korean regulatory process, it is an important milestone demonstrating the transition “from validation toward regulation.” That said, the design constraints of a single center and a comparatively small sample (*n* = 1106) should be considered when interpreting the results (see Section 5.3).

### 4.5. Reti-CVD Within the Broader Ecosystem of Retina-Based Approaches

Reti-CVD does not exist in isolation, and positioning it fairly requires situating it among competing and complementary retina-based approaches (Table 2). One line of work automates retinal-vessel-caliber measurement and derives CVD risk from it [19]; another predicts cardiovascular risk factors or risk directly from the image in an end-to-end manner [13]; retinal foundation models now provide transferable representations spanning multiple diseases, including cardiovascular endpoints [21]; and disease-group–specific models have been developed, for example in type 2 diabetes [34]. Against this backdrop, what distinguishes Reti-CVD is not a categorically different mechanism but the comparatively greater breadth of external validation and its progression along a regulatory pathway. We therefore treat it as an exemplar of the approach: useful for examining how such tools might be positioned and implemented, while recognizing that several of the points raised here apply to the wider field. Because the contextual literature was not screened in duplicate under a registered protocol, this overview may preferentially represent readily retrievable or positive approaches and should be read as illustrative rather than exhaustive.

A specific clinical group illustrates both the promise and the boundary of the current evidence. In an independent study (a separate algorithm of the same concept) in a type 2 diabetes cohort, retina-predicted risk was associated with major adverse cardiovascular events, showed discrimination comparable to the PCE, and improved when combined with a polygenic risk score [34]. This suggests the possibility of “unified screening” during diabetic retinal screening; however, because it used a separate algorithm rather than Reti-CVD itself (see the “output index” column of Table 1), Reti-CVD–specific validation in diabetic populations remains limited.

Recent device-specific studies add limited but relevant translational evidence. A prospective single-center acquisition study reported overall repeatability and reproducibility ICCs of 0.997 and 0.999, supporting measurement consistency but not prognostic or clinical utility [35]. A retrospective study in hypertensive retinopathy (*n* = 102) found risk-score separation by retinopathy severity and improved classification when combined with conventional models [36], while a 2026 short report explored cardiovascular risk assessment and statin-guidance implications in retinal vein occlusion [37]. These small, phenotype-specific observational studies do not establish that model-guided care improves outcomes.

## 5. Clinical Positioning

### 5.1. An Enhancer, Not a Replacement

The evidence to date positions Reti-CVD as a risk enhancer rather than a replacement for standard risk scores. In the UK Biobank study, adding Reti-CVD to QRISK3 improved discrimination, suggesting that the retina may carry additional information not fully captured by existing scores [22]. Given the guideline trend of recommending additional “risk-enhancing factors” in borderline/intermediate-risk groups [5,6], the natural role of Reti-CVD is to augment decision-making rather than to replace standard scores. In interpreting this positioning, three levels of evidence should be kept distinct: discrimination (how well the index ranks risk), reclassification (whether adding the index moves individuals across decision-relevant thresholds), and clinical outcome benefit (whether acting on the index improves patient-important outcomes). The current evidence for Reti-CVD speaks to the first two but not yet to the third.

### 5.2. Reclassification of Borderline/Intermediate-Risk Groups as the Core Indication

The point at which Reti-CVD shows the most compelling clinical value is the reclassification of borderline groups in which treatment decisions are uncertain. In the UK Biobank, among borderline-QRISK3 individuals, the actual 10-year risk of those reclassified as Reti-CVD high-risk was observed to exceed the intervention threshold (10%) [22]. This suggests a concrete use scenario of “non-invasively moving a patient in the gray zone of the standard score up one tier.” Conversely, in clearly low- or high-risk groups, the marginal utility of additional testing may be small, implying that targeted rather than indiscriminate application is reasonable. These observations remain hypothesis-generating. The specific clinical-utility claim—that Reti-CVD-guided reclassification of borderline patients changes management and improves patient-important outcomes—has not yet been subjected to a prospective trial designed to test and potentially refute it.

### 5.3. Relationship with Subclinical Atherosclerosis Markers (Cautious Interpretation)

The CMERC-HI study is of interest in that it evaluated Reti-CVD within the same analytic framework as established subclinical atherosclerosis markers such as CAC, CIMT, and baPWV. In that study, the discrimination of Reti-CVD was within a range broadly similar to these markers, and it retained an independent association even when several markers were included together in the model [24]. However, interpreting this observation as meaning that Reti-CVD is superior to existing markers warrants caution.

The reasons are as follows. First, this result was obtained in a comparatively small sample from a single center and a single cohort; it was not a direct comparison study designed and powered in advance to test superiority. Second, the confidence intervals of the reported discrimination differences overlap substantially, and some reference markers did not reach statistical significance in univariable analysis. Third, because subclinical markers differ in measurement protocol and clinical context, numerical comparison within a single cohort is difficult to generalize. Therefore, this review interprets the result as “exploratory, hypothesis-generating evidence suggesting that Reti-CVD may provide a level of information similar to established markers non-invasively,” and leaves any direct judgment of superiority to appropriately designed comparison studies.

### 5.4. Placement Scenarios Within the Care Pathway

**Figure 2:**
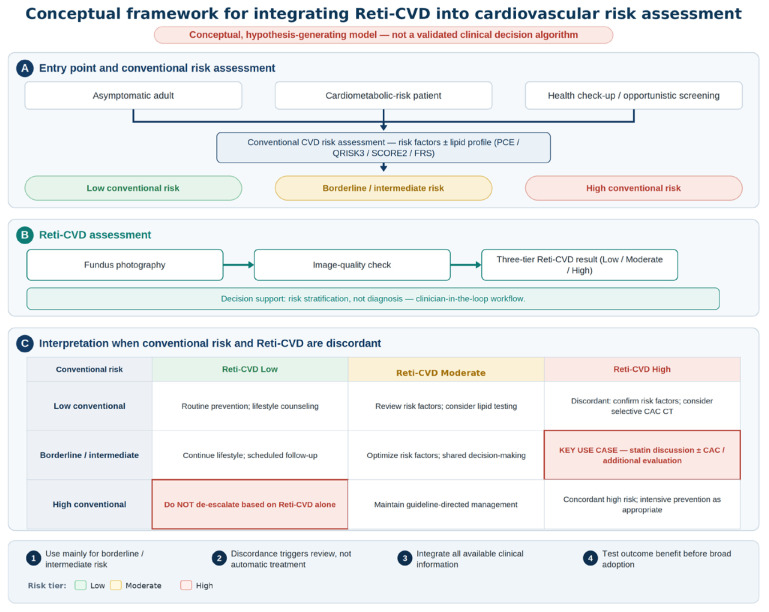
This is a conceptual, hypothesis-generating model, not a validated clinical decision algorithm: a proposed clinical decision framework for integrating Reti-CVD into cardiovascular risk assessment. The framework illustrates how a three-tier Reti-CVD result might be used alongside conventional risk assessment, with particular attention to actions when the two are discordant. It is a proposed, illustrative aid for shared decision-making and is not a validated, evidence-based treatment algorithm: the supporting evidence for Reti-CVD is observational, and no randomized or outcome-based intervention study has yet demonstrated that acting on these recommendations improves clinical outcomes. Accordingly, Reti-CVD should be used as a risk-enhancing, decision-support tool—not a stand-alone diagnostic test—and all clinical actions should integrate the totality of available information within shared decision-making. The framework is therefore intended to structure clinical discussion, not to justify initiating, withholding, or intensifying pharmacotherapy solely on the basis of Reti-CVD. Abbreviations as in Table 1; CAC CT, coronary artery calcium computed tomography.

In summary, the rational placement of Reti-CVD can be organized into three scenarios. First, it may serve as a first-line screening tool when blood sampling or laboratory access is limited. Second, it may serve as a second-line reclassification tool for patients judged borderline by standard scores. Third, it may be added opportunistically where fundus photography is already performed, such as diabetic retinal screening [34]. As illustrated in Panel A of Figure 2, these uses begin with a defined clinical entry point and conventional risk assessment; Panel B depicts retinal-image acquisition, image-quality review, and three-tier Reti-CVD output. Panel C operationalizes concordant and discordant results: borderline/intermediate conventional risk with high Reti-CVD may prompt additional evaluation and shared decision-making, whereas high conventional risk with low Reti-CVD should not prompt treatment de-escalation. These pathways are illustrative hypotheses, not validated treatment algorithms, and require outcome-based testing.

## 6. Implementation and Regulatory Perspectives

### 6.1. Regulatory Status: Organized by Jurisdiction

The academic name of the clinical tool addressed in this review is Reti-CVD, whereas the commercial software medical device is DrNoon for CVD (or the integrated European product DrNoon), manufactured by Mediwhale [25,27]. Table 3 distinguishes independently verifiable official information from manufacturer-reported status, based on sources checked through 10 July 2026.

A point requiring careful wording is the Korean market access. Under the official assessment-deferral listing, the retinal-image-based AI CVD risk assessment was available on a non-covered basis from 1 June 2023 through 31 May 2026 [38]. The pathway required patient explanation and non-reimbursed billing and did not constitute national health-insurance reimbursement. Because the designated period has ended, the post-deferral status should be rechecked against current NECA/HIRA or Ministry listings before any claim of continuing coverage or reimbursement.

In summary, Korean MFDS marketing authorization is independently verifiable [26], whereas the manufacturer reports CE MDR certification for the integrated European product [39] and an ongoing US FDA De Novo pathway [25]. The corresponding European certificate number and risk class were not independently identified in the public EUDAMED information reviewed [40]. The product previously entered Korean outpatient practice through a time-limited non-covered assessment-deferral pathway [38], which did not constitute national reimbursement. Jurisdiction-specific, source-qualified wording is therefore preferable to the generic term “regulatory-approved.”

### 6.2. Intended Use and Its Nature as Clinical Decision Support

According to the manufacturer’s materials, the tool’s purpose is to analyze a retinal photograph to assess and predict future cardiovascular disease risk, and its functional type is described as “retina-based coronary artery calcium score prediction” [25]. The European product materials describe the intended use more explicitly, defining it as software that “assists medical personnel” by analyzing a patient’s retinal image to detect eye diseases and estimate cardiovascular risk—aiding diagnosis for eye diseases while assessing the risk of future cardiovascular events [27]. That is, the European DrNoon is a combined product performing both eye-disease detection and CVD risk estimation, and the performance of its CVD-risk function is described as a Harrell C-index of 0.7518 [27], consistent with the value reported in the published validation study (0.751) [24].

These descriptions indicate that the tool provides risk stratification rather than a definitive diagnosis, and that it is a clinical decision-support tool premised on the clinician’s interpretation and decision rather than autonomous judgment. The manufacturer’s usage flow likewise comprises a clinician-in-the-loop structure: patient selection → clinician consultation → fundus photography → AI analysis → review of results [25]. Although some media use terms such as “autonomous” and “diagnostic,” these differ in tone from the manufacturer’s clinician-in-the-loop workflow and intended use. This review does not adopt them. This characterization of “risk stratification, not diagnosis” and “decision support, not autonomy” is important for setting clinical expectations and lines of responsibility.

### 6.3. Integration into the Clinical Workflow

Actual implementation requires designing the entire care flow beyond algorithm performance. The key elements are the dissemination of fundus cameras (especially non-mydriatic and handheld devices), the quality control of image capture by non-specialists, the linkage of automated results to the electronic medical record, and a referral pathway by which results lead to actual clinical action. That the manufacturer’s workflow is relatively simple (results within minutes after fundus photography) and designed for use in primary care is an advantage for integration [25]. However, unless a closed loop is in place, the clinical value of the risk information is limited. Such a loop connects results to the clinician’s judgment, patient education, and, where necessary, additional testing or treatment. Evaluation of this early clinical-adoption stage gains transparency when conducted according to reporting guidelines such as DECIDE-AI [41].

### 6.4. Equity, Generalizability, and Data Bias

Deep-learning imaging models are sensitive to the population and device distribution of the training data. Because the core validation of Reti-CVD was conducted mainly in East Asian and European-ancestry cohorts, generalizability to other ethnicities, diverse camera models, and different image-quality conditions requires further validation. Its non-invasive, low-cost nature has the potential to enhance access to risk assessment in resource-limited settings and thereby contribute to equity. At the same time, uncritical extension to unvalidated populations may lead to performance degradation and potential harm, so the confirmation of performance in the relevant population should be a prerequisite before application. Because multi-jurisdictional authorization does not automatically guarantee such generalizability, real-world performance monitoring in each application setting is important. Concretely, before broad application, the minimum evidence should include external validation in ancestries and healthcare settings beyond East Asian and European-ancestry cohorts, evaluation across multiple non-mydriatic and handheld camera models and image-quality strata, and prospective confirmation—or recalibration—of the low/moderate/high thresholds in each target population rather than reuse of cut-offs optimized in the derivation cohort. These implementation concerns are consistent with broader cautions regarding ethical deployment, clinical translation, reproducibility, and healthcare-algorithm bias in medical AI [42,43,44,45,46,47,48]; they also align with retinal vascular epidemiology showing that retinal-caliber associations can be population- and blood-pressure context dependent [49].

### 6.5. Cost, Access, and Reimbursement

Operationally, the tool’s key strengths are non-invasiveness, absence of radiation, short examination time, and compatibility with existing fundus-photography infrastructure. Compared with CT-based CAC, barriers related to equipment, radiation, and cost may be lower. In Korea, however, the product entered clinical practice through a time-limited non-covered assessment-deferral pathway ending 31 May 2026; this should not be equated with national reimbursement, and the post-deferral status requires current official confirmation. Whether practical advantages translate into clinical benefit and cost-effectiveness must be established by independent analyses and outcome-based studies.

## 7. Limitations and Evidence Gaps

While the evidence to date demonstrates the promise of the approach, the following gaps remain before broad clinical integration can be justified.

- **Concentration in one group/algorithm.** Much of the core validation was generated by the same research group using a single commercial algorithm, so independent external validation is lacking. This is the central caveat of the present evidence base and calls for caution from the standpoints of reproducibility and independence; it also means that a review centered on this tool must be read as an appraisal of an exemplar rather than of a mature, multi-vendor field.
- **Absence of randomized/outcome-based evidence.** Current observational findings are preliminary and hypothesis-generating. No trial has yet subjected the proposed clinical-utility claim—especially that Reti-CVD-guided reclassification of borderline patients changes treatment and reduces patient-important events—to a rigorous, potentially falsifying test. Predictive accuracy and reclassification therefore cannot be equated with successful clinical implementation or patient benefit. Accordingly, future evidence should separate SaMD clinical association, analytical/technical validation, and clinical performance, and prospective trials should follow AI-specific protocol and reporting guidance [50,51,52].
- **Need to distinguish output indices.** Some early evidence uses RetiCAC as the output index, which is only the conceptual foundation of Reti-CVD and should not be regarded as identical clinical evidence (Box 1). The frequent interchangeable use of the two names throughout the literature warrants interpretive caution.
- **Inter-cohort heterogeneity and indirect comparison.** UK Biobank, SEED, and CMERC-HI differ in population, outcome definition, and follow-up period, limiting direct comparison, and most comparisons with existing markers are indirect.
- **Possibility of selection bias.** Because this narrative review had no registered protocol and no independent duplicate screening, study selection may have favored readily retrievable, frequently cited, or positive reports and may have underrepresented negative or less visible contextual evidence, particularly in Section 4.5. Table 2 should therefore be interpreted as illustrative rather than exhaustive. This limitation was explicitly considered in the light of published quality-assessment guidance for narrative reviews [53].
- **Limitations of regulatory and reimbursement source information.** The manufacturer reported CE MDR certification in 2026 [39], but the corresponding certificate number and risk class were not independently identifiable in the public EUDAMED information reviewed [40]. Korea’s post-deferral market-access or reimbursement status after 31 May 2026 also remained uncertain [38]. These claims are therefore presented with explicit source attribution and require rechecking before publication.
- **Interpretability and data bias.** Explainability regarding which retinal features the model responds to is limited, and distribution bias in the training data may lead to equity issues. Standardized reporting and external validation are key means of mitigation [33]. Two related questions remain open. First, whether current post hoc explainability (for example, saliency mapping) is sufficient to support routine physician–patient communication, and justification of individual clinical decisions is uncertain, since such methods highlight influential image regions rather than verifiable biological mechanisms. Second, because the models learn from whole images, they may partly encode non-biological confounding signals—such as differences in image quality, camera hardware, and acquisition or processing pipelines across centers—rather than vascular biology alone; evaluation across devices and sites, with image-quality control and site-level analysis, is needed to separate genuine physiological signals from such artefacts.
- **Under-reported calibration and net clinical benefit.** The available validation evidence emphasizes discrimination (C-index or C-statistic) and relative risk, whereas calibration and net clinical benefit—which more directly govern real-world decisions—are reported inconsistently or not at all. Before implementation, model calibration (for example, calibration slope and intercept and observed-versus-predicted agreement across risk strata) and the added net benefit relative to standard scores (for example, by decision-curve analysis) should be assessed and reported in line with prediction-model reporting standards such as TRIPOD+AI [33]. Future studies should also report calibration and clinical net benefit using established calibration and decision-curve frameworks [54,55].
- **Potential harms of opportunistic screening.** The accessibility and low per-test burden of retinal screening should not be equated with an absence of harm. Applied opportunistically to broad, low-prevalence populations, the tool will generate false-positive results that can provoke anxiety, trigger downstream confirmatory testing, and consume finite clinical resources; the population-level balance of benefits and harms has not been established and would depend on the pre-test risk of the screened group and on the downstream management pathway. Targeted use in individuals for whom reclassification would change management is therefore more defensible than indiscriminate screening.
- **Generalizability.** Performance in settings outside the validated population and devices is uncertain, and confirmation in the relevant context is needed before application.

## 8. Future Research Directions

- **Independent multicenter prospective validation.** Prospective external validation in institutions, populations, and devices unrelated to the developing group is the priority task and should include diverse ethnicities and camera conditions. Transparent reporting should retain core TRIPOD principles together with TRIPOD+AI elements [33,56].
- **Outcome-based intervention studies.** Pragmatic prospective trials should predefine the management pathway triggered by Reti-CVD, treatment uptake, safety, and patient-important outcomes, and should be designed to test whether Reti-CVD-guided reclassification of borderline patients actually reduces events. This is the key step from predictive accuracy to clinical utility. Experience from retinal-AI clinical validation in diabetic retinopathy and retinal disease also illustrates that technical accuracy must be linked to a defined workflow and human–AI interaction before deployment [57,58].
- **Appropriately designed direct comparison studies.** Studies with prespecified power calculations and clear comparison designs are needed to evaluate fairly the relative value against established markers such as CAC. Such comparisons should include standard multivariable risk functions and established nontraditional risk-marker benchmarks, including CAC-based reclassification evidence [59,60].
- **Transparent description of the RetiCAC–Reti-CVD relationship.** Clearly disclosing the algorithmic relationship between the two output indices (whether recalibrated or redefined) and the rationale for threshold settings would reduce ambiguity in evidence interpretation.
- **Cost-effectiveness and reimbursement evidence.** Cost-effectiveness analyses for screening/primary-care adoption and jurisdiction-specific reimbursement evidence are prerequisites for dissemination.
- **Multimodal integration.** A multimodal approach combining the retinal index with clinical information, polygenic risk scores, and other oculomics markers is a promising route to improved discrimination [21,34].

## 9. Conclusions

Reti-CVD is a deep-learning index that stratifies CVD risk non-invasively from a retinal photograph and—among retina-based deep-learning approaches—is one of the most extensively externally validated and furthest advanced along a regulatory pathway. It developed from RetiCAC, which used CAC as a surrogate ground-truth label, and has been validated in multiple external cohorts and in a study supporting regulatory authorization. As the commercial product DrNoon for CVD, it holds Korean MFDS marketing authorization; the manufacturer reports CE MDR certification for the integrated European DrNoon product and US FDA authorization remains pending. In Korea, it previously entered outpatient practice through a time-limited non-covered assessment-deferral pathway. Current evidence supports positioning the tool as an enhancer to standard risk scores—particularly for borderline/intermediate-risk reclassification—rather than as a tool of established clinical utility.

This review’s contribution is therefore one of clinical positioning and implementation, using Reti-CVD as an exemplar of a broader, rapidly developing field rather than as a product endorsement. The concentration of evidence in a single research group and single algorithm, the absence of randomized/outcome-based evidence, the need to distinguish the RetiCAC and Reti-CVD output indices, inter-cohort heterogeneity, and uncertainties of equity and generalizability are challenges that must be resolved before broad clinical integration. The relative value against subclinical markers must be confirmed by appropriately designed direct comparison studies, and, above all, intervention studies are required to show whether risk reclassification improves clinical outcomes. When independent validation, outcome-based evidence, and cost-effectiveness data are accumulated, retina-based deep-learning risk stratification—exemplified here by Reti-CVD—has the potential to broaden access to cardiovascular primary prevention. Until a prospective intervention study demonstrates that Reti-CVD-guided reclassification improves patient-important outcomes, the transition from predictive performance to clinical implementation remains scientifically incomplete.

## Figures and Tables

**Table 2:** Reti-CVD positioned within the broader ecosystem of retina-based deep-learning approaches to cardiovascular risk.

Approach (Representative Work)	Input → Output Target	Validation Breadth	Regulatory/Translational Stage
End-to-end, CAC-trained score—Reti-CVD/RetiCAC [20,22,23,24]	Fundus photograph → three-tier CVD risk (trained on CAC label)	Multinational external cohorts + validation supporting regulatory authorization	MFDS authorization; manufacturer-reported CE MDR certification; time-limited non-covered use in Korea through May 2026; manufacturer-reported FDA De Novo pathway
Retinal-vessel-caliber DL system [19]	Fundus → automated vessel caliber → CVD risk	Multi-cohort	Research stage
Direct DL prediction of risk factors/risk [13]	Fundus → cardiovascular risk factors/risk	Development + validation	Research stage
Retinal foundation models (e.g., RETFound) [21]	Fundus → transferable representations (multi-disease, incl. CV)	Multi-task	Research stage
Disease-group–specific DL (type 2 diabetes) [34]	Fundus → CVD outcomes in T2D	Single cohort/program	Research stage

Representative examples are illustrative, not exhaustive. “Regulatory/translational stage” reflects source-qualified public information checked through 10 July 2026; manufacturer-reported items are identified as such in Section 6.1. This review treats Reti-CVD as an exemplar of the approach rather than as an endorsement of a specific product.

**Table 3:** Jurisdiction-specific regulatory and market-access status of DrNoon/Dr. Noon CVD (checked 10 July 2026).

Jurisdiction	Status and Source	Interpretation for this Review
Republic of Korea	MFDS marketing authorization: DrNoon for CVD, No. 22-513; initial approval 1 August 2022; validity through 31 July 2027 [25,26].	The assessment-deferral pathway permitted time-limited non-covered use from 1 June 2023 to 31 May 2026 [38]. This was not national reimbursement; post-deferral coverage/market-access status requires current official confirmation.
Europe	The manufacturer reported that the integrated DrNoon product (Dr. Noon CVD + Dr. Noon Fundus) obtained CE MDR certification in April 2026 [39].	The corresponding certificate number and risk class were not independently identifiable in the public EUDAMED information reviewed [40]; the MDR status is therefore presented as manufacturer-reported rather than independently registry-verified.
USA	The manufacturer reports Dr. Noon CVD as under the FDA De Novo pathway [25].	FDA authorization has not yet been reported; the product should not be described as “FDA-approved.”

## Data Availability

No new data were created or analyzed in this study. All sources cited are publicly available.

## References

[1] Roth G.A., Mensah G.A., Johnson C.O., Addolorato G., Ammirati E., Baddour L.M., Barengo N.C., Beaton A.Z., Benjamin E.J., Benziger C.P. (2020). Global burden of cardiovascular diseases and risk factors, 1990–2019. J. Am. Coll. Cardiol..

[2] Hippisley-Cox J., Coupland C., Brindle P. (2017). Development and validation of QRISK3 risk prediction algorithms to estimate future risk of cardiovascular disease: Prospective cohort study. BMJ.

[3] SCORE2 Working Group and ESC Cardiovascular Risk Collaboration (2021). SCORE2 risk prediction algorithms: New models to estimate 10-year risk of cardiovascular disease in Europe. Eur. Heart J..

[4] Goff D.C., Lloyd-Jones D.M., Bennett G., Coady S., D’Agostino R.B., Gibbons R., Greenland P., Lackland D.T., Levy D., O’Donnell C.J. (2014). 2013 ACC/AHA guideline on the assessment of cardiovascular risk. Circulation.

[5] Arnett D.K., Blumenthal R.S., Albert M.A., Buroker A.B., Goldberger Z.D., Hahn E.J., Himmelfarb C.D., Khera A., Lloyd-Jones D., McEvoy J.W. (2019). 2019 ACC/AHA guideline on the primary prevention of cardiovascular disease. Circulation.

[6] Visseren F.L.J., Mach F., Smulders Y.M., Carballo D., Koskinas K.C., Bäck M., Benetos A., Biffi A., Boavida J.-M., Capodanno D. (2021). 2021 ESC guidelines on cardiovascular disease prevention in clinical practice. Eur. Heart J..

[7] Detrano R., Guerci A.D., Carr J.J., Bild D.E., Burke G.L., Folsom A.R., Liu K., Shea S., Szklo M., Bluemke D.A. (2008). Coronary calcium as a predictor of coronary events in four racial or ethnic groups. N. Engl. J. Med..

[8] Greenland P., Blaha M.J., Budoff M.J., Erbel R., Watson K.E. (2018). Coronary calcium score and cardiovascular risk. J. Am. Coll. Cardiol..

[9] Wong T.Y., Klein R., Sharrett A.R., Duncan B.B., Couper D.J., Tielsch J.M., Klein B.E.K., Hubbard L.D. (2002). Retinal arteriolar narrowing and risk of coronary heart disease in men and women: The Atherosclerosis Risk in Communities Study. JAMA.

[10] Wong T.Y., Mitchell P. (2004). Hypertensive retinopathy. N. Engl. J. Med..

[11] McGeechan K., Liew G., Macaskill P., Irwig L., Klein R., Klein B.E.K., Wang J.J., Mitchell P., Vingerling J.R., de Jong P.T.V.M. (2009). Prediction of incident stroke events based on retinal vessel caliber: A systematic review and individual-participant meta-analysis. Am. J. Epidemiol..

[12] Seidelmann S.B., Claggett B., Bravo P.E., Gupta A., Farhad H., Klein B.E., Klein R., Di Carli M., Solomon S.D. (2016). Retinal vessel calibers in predicting long-term cardiovascular outcomes: The Atherosclerosis Risk in Communities Study. Circulation.

[13] Poplin R., Varadarajan A.V., Blumer K., Liu Y., McConnell M.V., Corrado G.S., Peng L., Webster D.R. (2018). Prediction of cardiovascular risk factors from retinal fundus photographs via deep learning. Nat. Biomed. Eng..

[14] Ghenciu L.A., Dima M., Stoicescu E.R., Iacob R., Boru C., Hațegan O.A. (2024). Retinal imaging-based oculomics: Artificial intelligence as a tool in the diagnosis of cardiovascular and metabolic diseases. Biomedicines.

[15] Bisen J.B., Sikora H., Aneja A., Shah S.J., Mirza R.G. (2025). Retinal imaging as a window into cardiovascular health: Towards harnessing retinal analytics for precision cardiovascular medicine. J. Cardiovasc. Dev. Dis..

[16] Wagner S.K., Fu D.J., Faes L., Liu X., Huemer J., Khalid H., Ferraz D., Korot E., Kelly C., Balaskas K. (2020). Insights into systemic disease through retinal imaging-based oculomics. Transl. Vis. Sci. Technol..

[17] Gulshan V., Peng L., Coram M., Stumpe M.C., Wu D., Narayanaswamy A., Venugopalan S., Widner K., Madams T., Cuadros J. (2016). Development and validation of a deep learning algorithm for detection of diabetic retinopathy in retinal fundus photographs. JAMA.

[18] Ting D.S.W., Cheung C.Y.-L., Lim G., Tan G.S.W., Quang N.D., Gan A., Hamzah H., Garcia-Franco R., Yeo I.Y.S., Lee S.Y. (2017). Development and validation of a deep learning system for diabetic retinopathy and related eye diseases using retinal images from multiethnic populations with diabetes. JAMA.

[19] Cheung C.Y., Xu D., Cheng C.-Y., Sabanayagam C., Tham Y.-C., Yu M., Rim T.H., Chai C.Y., Gopinath B., Mitchell P. (2021). A deep-learning system for the assessment of cardiovascular disease risk via the measurement of retinal-vessel calibre. Nat. Biomed. Eng..

[20] Rim T.H., Lee C.J., Tham Y.-C., Cheung N., Yu M., Lee G., Kim Y., Ting D.S.W., Chong C.C.Y., Choi Y.S. (2021). Deep-learning-based cardiovascular risk stratification using coronary artery calcium scores predicted from retinal photographs. Lancet Digit. Health.

[21] Zhou Y., Chia M.A., Wagner S.K., Ayhan M.S., Williamson D.J., Struyven R.R., Liu T., Xu M., Lozano M.G., Woodward-Court P. (2023). A foundation model for generalizable disease detection from retinal images. Nature.

[22] Tseng R.M.W.W., Rim T.H., Shantsila E., Yi J.K., Park S., Kim S.S., Lee C.J., Thakur S., Nusinovici S., Peng Q. (2023). Validation of a deep-learning-based retinal biomarker (Reti-CVD) in the prediction of cardiovascular disease: Data from UK Biobank. BMC Med..

[23] Yi J.K., Rim T.H., Park S., Kim S.S., Kim H.C., Lee C.J., Kim H., Lee G., Lim J.S.G., Tan Y.Y. (2023). Cardiovascular disease risk assessment using a deep-learning-based retinal biomarker: A comparison with existing risk scores. Eur. Heart J. Digit. Health.

[24] Lee C.J., Rim T.H., Kang H.G., Yi J.K., Lee G., Yu M., Park S.-H., Hwang J.-T., Tham Y.-C., Wong T.Y. (2024). Pivotal trial of a deep-learning-based retinal biomarker (Reti-CVD) in the prediction of cardiovascular disease: Data from CMERC-HI. J. Am. Med. Inform. Assoc..

[25] Mediwhale Inc Dr. Noon CVD (Product Name: DrNoon for CVD): Product and Regulatory Information. https://mediwhale.com/drnoon-cvd/.

[26] Ministry of Food and Drug Safety (MFDS), Republic of Korea Medical Device Marketing Authorization Information: DrNoon for CVD (Authorization No. 제허 22-513호). https://emedi.mfds.go.kr.

[27] Mediwhale Inc. DrNoon (Europe): Intended Use and Performance. https://www.mediwhale-drnoon.com/.

[28] Li L.Y., Isaksen A.A., Lebiecka-Johansen B., Funck K., Thambawita V., Byberg S., Andersen T.H., Norgaard O., Hulman A. (2024). Prediction of cardiovascular markers and diseases using retinal fundus images and deep learning: A systematic scoping review. Eur. Heart J. Digit. Health.

[29] Whiting P.F., Rutjes A.W.S., Westwood M.E., Mallett S., Deeks J.J., Reitsma J.B., Leeflang M.M.G., Sterne J.A.C., Bossuyt P.M.M., QUADAS-2 Group (2011). QUADAS-2: A revised tool for the quality assessment of diagnostic accuracy studies. Ann. Intern. Med..

[30] Wolff R.F., Moons K.G., Riley R., Whiting P.F., Westwood M., Collins G.S., Reitsma J.B., Kleijnen J., Mallett S., for the PROBAST Group (2019). PROBAST: A tool to assess the risk of bias and applicability of prediction model studies. Ann. Intern. Med..

[31] Rim T.H., Lee G., Kim Y., Tham Y.-C., Lee C.J., Baik S.J., Kim Y.A., Yu M., Deshmukh M., Lee B.K. (2020). Prediction of systemic biomarkers from retinal photographs: Development and validation of deep-learning algorithms. Lancet Digit. Health.

[32] Son J., Shin J.Y., Chun E.J., Jung K.H., Park K.H., Park S.J. (2020). Predicting high coronary artery calcium score from retinal fundus images with deep learning algorithms. Transl. Vis. Sci. Technol..

[33] Collins G.S., Moons K.G.M., Dhiman P., Riley R.D., Beam A.L., Van Calster B., Ghassemi M., Liu X., Reitsma J.B., van Smeden M. (2024). TRIPOD+AI statement: Updated guidance for reporting clinical prediction models that use regression or machine learning methods. BMJ.

[34] Syed M.G., Trucco E., Mookiah M.R.K., Lang C.C., McCrimmon R.J., Palmer C.N.A., Pearson E.R., Doney A.S.F., Mordi I.R. (2025). Deep-learning prediction of cardiovascular outcomes from routine retinal images in individuals with type 2 diabetes. Cardiovasc. Diabetol..

[35] Hong R.K., Kim M., Hong E.H., Kang M.H., Shin Y.U., Park H.-C., Hwang S. (2026). Quantifying the repeatability and reproducibility of Dr. Noon CVD, AI software as medical device for cardiovascular risk assessment via retinal imaging. Can. J. Ophthalmol..

[36] Nam D., Jang Y.-H., Lee Y., Seo J., Thakur S., Nusinovici S., Kim M., Shin Y.U., Park H.-C., Hwang S. (2026). Clinical utility of an AI-based retinal imaging model for cardiovascular risk prediction in hypertensive retinopathy. Can. J. Ophthalmol..

[37] Nam D., Jang Y.-H., Ryu S.J., Thakur S., Nusinovici S., Park J., Kim M., Hwang S. (2026). Artificial intelligence based retinal imaging for cardiovascular risk and statin guidance in retinal vein occlusion. Am. J. Prev. Cardiol..

[38] Ministry of Health and Welfare, Republic of Korea Evaluation-Deferred New Health Technologies: Retinal-Image–Based Artificial-Intelligence Cardiovascular Risk Assessment Software (DrNoon/Reti-CVD), Designated Period 1 June 2023–31 May 2026. https://law.go.kr.

[39] Mediwhale Inc. (2026). Mediwhale Wins EU CE MDR Approval for Retinal AI Cardiovascular Risk Tool. Mediwhale.

[40] European Commission European Database on Medical Devices (EUDAMED), Public Website. https://ec.europa.eu/tools/eudamed/.

[41] Vasey B., Nagendran M., Campbell B., Clifton D.A., Collins G.S., Denaxas S., Denniston A.K., Faes L., Geerts B., Ibrahim M. (2022). Reporting guideline for the early-stage clinical evaluation of decision support systems driven by artificial intelligence: DECIDE-AI. Nat. Med..

[42] Char D.S., Shah N.H., Magnus D. (2018). Implementing machine learning in health care—Addressing ethical challenges. N. Engl. J. Med..

[43] Rajkomar A., Dean J., Kohane I. (2019). Machine learning in medicine. N. Engl. J. Med..

[44] Topol E.J. (2019). High-performance medicine: The convergence of human and artificial intelligence. Nat. Med..

[45] Kelly C.J., Karthikesalingam A., Suleyman M., Corrado G., King D. (2019). Key challenges for delivering clinical impact with artificial intelligence. BMC Med..

[46] Wiens J., Saria S., Sendak M., Ghassemi M., Liu V.X., Doshi-Velez F., Jung K., Heller K., Kale D., Saeed M. (2019). Do no harm: A roadmap for responsible machine learning for health care. Nat. Med..

[47] Vollmer S., A Mateen B., Bohner G., Király F.J., Ghani R., Jonsson P., Cumbers S., Jonas A., McAllister K.S.L., Myles P. (2020). Machine learning and artificial intelligence research for patient benefit: 20 critical questions on transparency, replicability, ethics, and effectiveness. BMJ.

[48] Obermeyer Z., Powers B., Vogeli C., Mullainathan S. (2019). Dissecting racial bias in an algorithm used to manage the health of populations. Science.

[49] Ding J., Wai K.L., McGeechan K., Ikram M.K., Kawasaki R., Xie J., Klein R., Klein B.B., Cotch M.F., Wang J.J. (2014). Retinal vascular caliber and the development of hypertension: A meta-analysis of individual participant data. J. Hypertens..

[50] International Medical Device Regulators Forum (IMDRF) (2017). Software as a Medical Device (SaMD): Clinical Evaluation; IMDRF/SaMD WG/N41FINAL:2017. https://www.imdrf.org/documents/software-medical-device-samd-clinical-evaluation.

[51] Liu X., Rivera S.C., Moher D., Calvert M.J., Denniston A.K., SPIRIT-AI and CONSORT-AI Working Group (2020). Reporting guidelines for clinical trial reports for interventions involving artificial intelligence: The CONSORT-AI extension. Nat. Med..

[52] Rivera S.C., Liu X., Chan A.-W., Denniston A.K., Calvert M.J., SPIRIT-AI and CONSORT-AI Working Group (2020). Guidelines for clinical trial protocols for interventions involving artificial intelligence: The SPIRIT-AI extension. Nat. Med..

[53] Baethge C., Goldbeck-Wood S., Mertens S. (2019). SANRA—A scale for the quality assessment of narrative review articles. Res. Integr. Peer Rev..

[54] Van Calster B., McLernon D.J., van Smeden M., Wynants L., Steyerberg E.W., Topic Group ‘Evaluating Diagnostic Tests and Prediction Models’ of the STRATOS Initiative (2019). Calibration: The Achilles heel of predictive analytics. BMC Med..

[55] Vickers A.J., Elkin E.B. (2006). Decision curve analysis: A novel method for evaluating prediction models. Med. Decis. Mak..

[56] Collins G.S., Reitsma J.B., Altman D.G., Moons K.G.M. (2015). Transparent reporting of a multivariable prediction model for individual prognosis or diagnosis (TRIPOD): The TRIPOD statement. Ann. Intern. Med..

[57] Abràmoff M.D., Lavin P.T., Birch M., Shah N., Folk J.C. (2018). Pivotal trial of an autonomous AI-based diagnostic system for detection of diabetic retinopathy in primary care offices. npj Digit. Med..

[58] De Fauw J., Ledsam J.R., Romera-Paredes B., Nikolov S., Tomasev N., Blackwell S., Askham H., Glorot X., O’donoghue B., Visentin D. (2018). Clinically applicable deep learning for diagnosis and referral in retinal disease. Nat. Med..

[59] D’Agostino R.B., Vasan R.S., Pencina M.J., Wolf P.A., Cobain M., Massaro J.M., Kannel W.B. (2008). General cardiovascular risk profile for use in primary care: The Framingham Heart Study. Circulation.

[60] Yeboah J., McClelland R.L., Polonsky T.S., Burke G.L., Sibley C.T., O’lEary D., Carr J.J., Goff D.C., Greenland P., Herrington D.M. (2012). Comparison of novel risk markers for improvement in cardiovascular risk assessment in intermediate-risk individuals. JAMA.

